# Preparation and Characterization of Hydroxyapatite from Eggshells via a Basic Route Using Attritor Milling

**DOI:** 10.3390/nano16150899

**Published:** 2026-07-23

**Authors:** Boglárka Almássy, Katalin Balázsi, Csaba Balázsi

**Affiliations:** 1 Doctoral School of Materials Science and Technologies, Óbuda University, Bécsi Str. 96/B, 1030 Budapest, Hungary; almassy.boglarka@ek.hun-ren.hu; 2 HUN-REN Centre for Energy Research, Konkoly-Thege Rd. 29-33, H-1121 Budapest, Hungary; balazsi.katalin@ek.hun-ren.hu

**Keywords:** eggshell, attrition milling, basic route, bone graft

## Abstract

In this study, pure hydroxyapatite (HAp) was successfully produced by using eggshells. The eggshells were collected locally and calcined to get CaO from them. The CaO powder was reacted with diammonium hydrogen phosphate in a mechanochemical method using attritor milling. A portion of the synthesized samples was subjected to a second calcination process at 900 °C to investigate the thermal effects on the material. The structures of the samples were investigated by scanning electron microscopy, X-ray diffraction, and infrared spectroscopy. The as-prepared HAp appeared to be nanocrystalline with low-intensity reflections, which transformed into a highly crystalline hexagonal phase after heat treatment, as revealed by XRD analysis. Quantitative analysis revealed the thermal evolution of the secondary Ca(OH)_2_ phase, due to the thermal decomposition into CaO without causing HAp decomposition into tricalcium phosphates. FTIR analysis showed characteristic phosphate bands for both samples, but the calcined sample displayed sharper peaks and a clear loss of residual water and carbonates. SEM observations also highlighted the major morphological transformation. The highly aggregated as-prepared nanoparticles formed larger, well-defined grains. Notably, the calcined sample also exhibited a rough, textured surface with a macroporous network with interconnected channels. EDS analysis confirmed a Ca–P–O-rich composition, where the elevated Ca/P ratio (Ca/P = 2.28) suggested the presence of secondary calcium-rich phases. These structural, chemical, and morphological characteristics suggest that eggshell-derived HAp, with or without a second heat treatment, has high potential and may be optimized for different applications in bone tissue engineering. However, biological performance was not evaluated in this study.

## 1. Introduction

**Figure 1:**
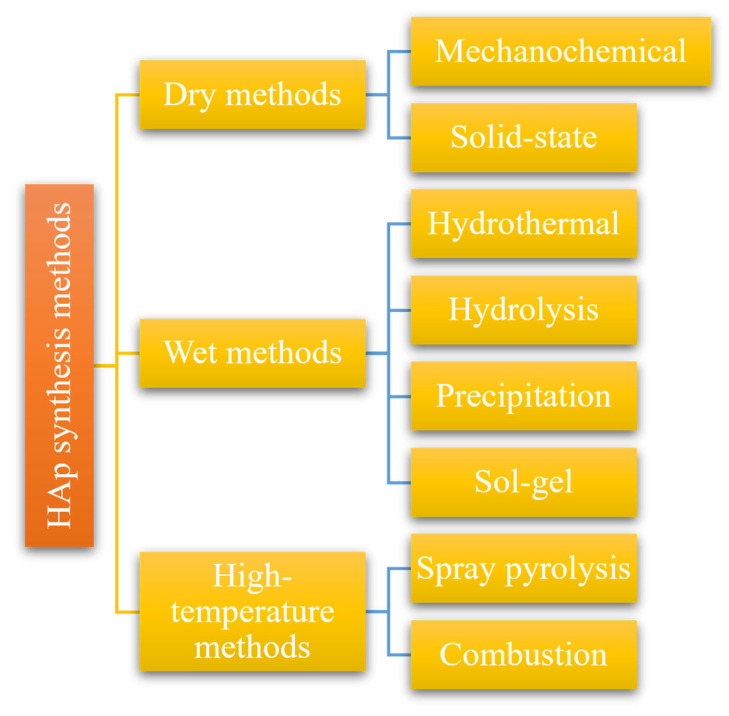
Synthesis methods of Hydroxyapatite (HAp).

The increasing lifespan of modern societies has led to a growing demand for effective bone repair and replacement procedures. Hydroxyapatite (HAp), with the chemical formula Ca_10_(PO_4_)_6_(OH)_2_, is the primary inorganic constituent of bone tissue [1]. Due to its excellent biocompatibility and bioactivity [2,3], HAp has been extensively studied and widely employed as a bone substitute material in various biomedical applications, including implant coatings, porous granules, synthetic bone grafts, and hard tissue scaffolds [4,5]. The history of HAp dates back to the early 19th century. At that time, researchers found out that HAp is the major mineral component of the human bone. Implanted HAp showed excellent biocompatibility and the ability to integrate with the surrounding bone tissue [6], which made HAp one of the most interesting materials in the bone replacement field, from dental to orthopedic applications. For standardized material, the development of the synthesis was necessary. In the 1980s, researchers successfully synthesized and characterized HAp with high precision [7]. Since then, different synthesis possibilities have been published by researchers in order to make the perfect material for the exact application, shortening the process time, finding environmentally optimal methods, etc. HAp can be synthesized through various methods, including dry processes (such as mechanochemical and solid-state methods), wet methods (including hydrothermal, hydrolysis, precipitation, sol–gel) [8,9], and high-temperature processes (such as spray pyrolysis and combustion) [10]. These techniques are shown in Figure 1.

In dry methods, the solid initial reactants are mixed and calcined at high temperatures to form HAp. In the solid-state method, the reactants are simply mixed and then calcined, whereas the mechanochemical process utilizes mechanical energy by grinding the precursors (often in a ball mill). This approach leads to a well-defined structure and offers good control over the product [11].

Through wet methods, the powder morphology and particle size are highly controllable. There are various techniques, such as hydrothermal, hydrolysis, precipitation, and sol–gel methods. Compared to dry methods, in wet processes, the calcium and phosphate precursors are dissolved in a solution (using different solvents and reaction temperatures) and react chemically to form HAp.

The hydrothermal method involves the reaction of precursors in an aqueous medium under elevated temperature and pressure, conditions that facilitate crystal growth and improve the crystallinity of the final product. This approach enables the synthesis of stoichiometric hydroxyapatite (HAp) with well-defined crystal morphologies [12]. Using a hydrothermal process, ref. [13] synthesized high-purity HAp from eggshell-derived CaO and diammonium hydrogen phosphate, obtaining hexagonal nanoparticles with a purity of up to 99.5%. Despite its excellent crystallinity and purity, the hydrothermal method requires an extended reaction time of 48 h and specialized high-pressure equipment, which limits its cost-effectiveness and scalability.

In the hydrolysis technique, HAp is prepared through the hydrolysis of other calcium phosphates (CaP), such as dicalcium phosphate anhydrous (DCPA) or tricalcium phosphate (TCP). These CaP salts or acidic phases are thermodynamically less stable at higher pH levels (typically six or above), leading to their transformation into the more stable HAp phase. This approach is strongly dependent on the pH value and temperature, which allows fine control, but on the other hand, it requires optimization [14]. It should be noted that HAp crystal formation in other wet methods often proceeds through similar intermediate phases [15].

The chemical precipitation method is the most common and simplest way to synthesize HAp. It is usually conducted at temperatures ranging from room temperature to the boiling point of water, and at pH values higher than 4.2, where HAp is the least soluble CaP phase [16,17]. In this technique, various calcium and phosphate precursors can be used; for example, calcium hydroxide or calcium nitrate serves as the Ca^2+^ source, while orthophosphoric acid or diammonium hydrogen phosphate serves as the PO_4_^3−^ source. One reagent is added dropwise under continuous stirring during the reaction to maintain the Ca/P ratio at the stoichiometric value of 1.67 [18]. Following the reaction, the suspension can be dried and crushed into a powder immediately, or aged at atmospheric pressure. Usually, powders prepared by simple precipitation are non-stoichiometric and poorly crystallized. To increase crystallinity and phase purity, the reaction should be conducted at high pH, high temperature, or both [14]. In another study, crystalline HAp nanoparticles were prepared by direct precipitation using eggshell-derived CaO and DHP as precursor materials. The resulting HAp consisted of agglomerated rod-like nanostructures with a specific surface area of 16.70 m^2^/g [19].

The sol–gel technique is suitable for synthesizing high-purity hydroxyapatite (HAp) at relatively low temperatures. The process begins with the preparation of a colloidal suspension (the sol) by dissolving calcium and phosphorus precursors in a solvent, where they undergo hydrolysis and polycondensation. Over time, the sol transitions into a porous network (the gel). The method was named after these two stages. After aging and drying to remove solvents, the resulting material is calcined (typically between 600 °C and 900 °C) to eliminate organic residues and promote the crystallization of the HAp phase. This process is highly valued in biomedical fields because it allows for molecular-level homogeneity at the stoichiometric Ca/P ratio of 1.67. Furthermore, the nanoscale HAp crystalline structure produced by this technique has been reported to be remarkably similar to natural human bone apatite [19,20].

High-temperature processes operate at high temperatures to decompose or partially combust the precursors. High-temperature processes are typically performed using two techniques: spray pyrolysis and combustion.

In the spray pyrolysis method, precursor solutions are sprayed into a flame or the hot zone of an electric furnace using an ultrasonic generator. A reaction between the generated vapors and gases then occurs at high temperatures to form HAp. The intense heat leads to the complete evaporation of the precursors, followed by the growth of nanoparticles in the gas phase. The final particle size is strongly dependent on the size of the initial droplets [21].

The combustion synthesis method relies on a rapid, self-sustaining exothermic redox reaction between oxidants (such as calcium nitrate and HNO_3_) and an organic fuel (e.g., glycine, urea, or citric acid) in the liquid phase. To initiate the oxidation, Ca(NO_3_)_2_ and (NH_4_)_2_HPO_4_ are mixed, and concentrated HNO_3_ is added, followed by the incorporation of fuels into the solution. The reaction is triggered by heating the mixture in a furnace at approximately 300 °C. During combustion, a sudden spike to a maximum flame temperature occurs. Fast cooling of the mixture is necessary to maximize nucleation and prevent further particle growth. The resulting material is typically characterized as agglomerates of very fine, porous particles [22].

Furthermore, environmentally friendly alternatives are gaining popularity, which utilize biowaste materials like bone, fish scales, mussel shells, and eggshells [23,24].

Plant-derived agricultural by-products have attracted increasing attention as sustainable precursors for hydroxyapatite synthesis. However, their broader application remains limited by inefficient extraction protocols and the compositional variability of plant-derived materials [25].

Mammalian sources are highly valued due to their compositional similarity to human bone, promoting excellent osteoconductivity and tissue regeneration. Organic components (collagen, fats) are removed from the bones via calcination or alkaline hydrolysis, leading to a pure inorganic HAp scaffold. Microwave-assisted calcination is an emerging technique to improve efficiency and scalability. It should be noted that these sources specifically require rigorous purification to eliminate immunogenic substances and prevent disease transmission [26].

Fish bones and scales are typically calcined, whereas seashells, due to their high CaCO_3_ content, are used as calcium precursors and usually converted into HAp via chemical precipitation or hydrothermal methods. Marine-derived HAp naturally contains trace elements (magnesium, strontium, sodium, fluorine) that improve the osseointegration of bones and teeth. The biological performance depends on the specific marine source; therefore, the optimization of extraction techniques is needed [27].

Eggshells (avian sources) are an abundant and low-cost raw material, which, if untreated, can contribute to environmental pollution and microbial contamination. Owing to their high calcium carbonate (CaCO_3_) content (91–94%) [28] and trace minerals such as magnesium and strontium, which are beneficial for bone formation [29], eggshells represent a promising and sustainable precursor for HAp synthesis [30]. After heat treatment, the calcium precursor is reacted with a phosphorus source using chemical precipitation, sol–gel methods, microwave-assisted synthesis, or mechanochemical methods [31]. These approaches can produce nano-sized HAp particles that exhibit high cell adhesion. On the other hand, process-to-process variability and inconsistent shell compositions make standardized production difficult.

Recent advances in sustainable chemistry have emphasized the importance of efficient element utilization, resource availability, and environmentally benign synthetic pathways in the development of modern chemical processes [32]. Efficient conversion of such waste materials into hydroxyapatite with controlled physicochemical properties remains challenging, particularly when aiming for nanoscale products with reproducible characteristics.

Recent reviews have highlighted the growing interest in developing simple, scalable, and environmentally sustainable synthesis strategies for producing high-purity nanosized hydroxyapatite with controlled physicochemical properties. In particular, the utilization of bio-derived calcium sources and green synthesis routes has emerged as a promising approach toward sustainable biomaterial production [33].

In this work, we create a novel technique for eggshell-derived HAp synthesis by using attritor milling combined with diammonium hydrogen phosphate (DAP) as the phosphate source. Although attritor milling has been previously applied in our laboratory for HAp production [34,35], its combination with DAP has not been extensively investigated. Attritor milling provides an effective mechanochemical route for synthesizing hydroxyapatite under the selected processing conditions. The synthesized powders were characterized by scanning electron microscopy (SEM), X-ray diffraction (XRD), and Fourier-transform infrared spectroscopy (FTIR) to evaluate their key physicochemical properties, which are critical for their potential application in bone repair and replacement [36,37].

## 2. Experimental Section

### 2.1. Materials and Methods

Eggshells were collected locally. They were mechanically crushed first and then washed with deionized water to remove any impurities. The cleaned eggshells were calcined in an air atmosphere at 900 °C for 10 h [38,39]. In the first 30 min of the calcination, the organic constituents were thermally decomposed. After that, the calcium carbonate (CaCO_3_) in the eggshell transformed into calcium oxide (CaO). The resulting CaO was the primary calcium precursor for the hydroxyapatite synthesis.

The HAp synthesis was performed using a Szegvari Attritor System (Model DM01, Union Process, Akron, OH, USA). The starting materials included the prepared calcium oxide (CaO) and analytical-grade diammonium hydrogen orthophosphate (≥98%, AnalaR NORMAPUR® ACS, Reag. Ph. Eur.; VWR Chemicals, Leuven, Belgium) (NH_4_)_2_HPO_4_, as the phosphate precursor, which were dispersed in deionized water. The milling process was conducted with zirconia tanks and balls. The attritor was operated at a rotational speed of 2000 RPM (27.45 Hz) for 5 h. The stoichiometric reaction for the synthesis is as follows:

10CaO + 6(NH_4_)_2_ HPO_4_ → Ca_10_ (PO_4_)_6_ (OH)_2_ + 12NH_3_ + 8H_2_O

After milling, the resulting powder was divided into two samples: HAp_1 (The as-milled powder) and HAp_900 (half of the as-milled sample was heat-treated in an air atmosphere at 900 °C for 1 h). The parameters for both samples are summarized in Table 1.

### 2.2. Characterization Methods

The crystallographic phases of powders were identified by using X-ray diffractometry (XRD) with a D8 Discover X-ray diffractometer (Bruker AXS, Karlsruhe, Germany), which was equipped with a Göbel mirror and a scintillation detector. The analysis utilized Cu Kα radiation (λ = 1.5406 Å). The X-ray beam dimensions were 1 mm by 5 mm, with a 2θ step size of 0.02° and a measurement duration of 3 s per step. For phase identification, quantitative analysis, and crystallite size determination, we utilized the Diffrac^plus^ EVA software (Version 11.0.0.3, Release 2005; Bruker AXS, Karlsruhe, Germany) incorporating built-in full pattern matching.

Fourier transform infrared spectra were recorded using a spectrophotometer (FTIR) (Spectrum Two, Perkin Elmer, Shelton, CT, USA) with a Diamond ATR unit. The thick films were directly introduced into the ATR accessory, and consistent pressure was applied to all samples. The FTIR spectra were recorded in the range from 4000 to 450 cm^−1^, with 8 scans and a 4 cm^−1^ resolution. The data analysis was performed with the Spectrum IR 10.7.2.1630 software.

The morphological analysis of the powders was performed using a Thermo Fisher Scios2 DualBeam scanning electron microscope (Thermo Fisher Scientific, Waltham, MA, USA) in conjunction with an Energy Dispersive X-ray Spectrometry (EDS) X-MAX-20 EDS detector (Oxford Instruments, Abingdon, UK). The analysis was conducted using AZtecOne 6.1 SP3 software (Oxford Instruments), with the EDS spectrum recorded at an accelerating voltage of 8 keV.

## 3. Results and Discussion

### 3.1. X-Ray Diffractometry (XRD)

**Figure 2:**
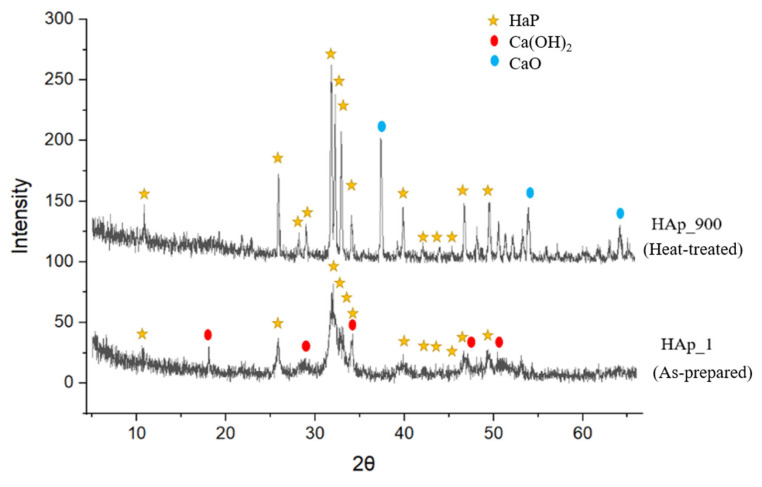
XRD characteristic patterns of the samples.

The diffraction pattern of the as-prepared HAp_1 sample (Figure 2) showed relatively low-intensity and broadened peaks, indicating the presence of nanocrystalline hydroxyapatite. The characteristic reflections observed at approximately 25.9°, 31.8°, 32.2°, 32.9°, 34.0°, 39.8°, 46.7°, and 49.5° (2θ) can be assigned to the (002), (211), (112), (300), (202), (310), (222), and (213) planes of hexagonal hydroxyapatite, in agreement with the standard JCPDS card 84-1998.

After calcination at 900 °C, the diffraction peaks became sharper and more intense, indicating an increase in crystallinity and crystal growth. This observation is also supported by the crystallite size values calculated using the Debye-Scherrer equation (Table 2) as follows:

$$D=\frac{k\cdot \lambda }{\beta \cdot \cos\theta }$$

where k is the Scherrer constant (describes the shape of the particle and its value), λ is the wavelength of the used X-ray beam (1.54, 184 Å), β corresponds to the full width at half maximum of the diffraction peak, expressed in radians (FWHM), and θ is the Bragg angle [40,41].

The calculated values showed an increase in HAp crystallite size from 123 Å in the HAp_1 sample to 560 Å in the HAp_900 sample. The broad peaks of the as-prepared sample and the increase in crystallite size after calcination are consistent with the behavior typically reported for hydroxyapatite from biogenic calcium sources. Similar behavior has been reported in previous studies as well. By using eggshells as calcium sources, ref. [42] reported a crystallite size of approximately 35 nm in synthesized hydroxyapatite, together with an increase in crystallinity and XRD peak sharpening with increasing temperature. Likewise, ref. [43] investigated pig bone-derived precursors and observed a clear temperature-dependent increase in crystallite size during hydroxyapatite formation.

At human body temperature (approximately 37 °C), stoichiometric hydroxyapatite is considered the thermodynamically stable calcium phosphate phase and has excellent chemical stability and biocompatibility, which explains its widespread use in bone tissue engineering and implant coatings [44,45]. The in vivo stability and dissolution behavior of hydroxyapatite are primarily influenced by factors such as crystallinity, crystal size, porosity, and the presence of ionic substitutions (e.g., carbonate, magnesium, or sodium) [46].

Quantitative phase analysis revealed that the HAp_1 sample contained 94.8% hydroxyapatite and 5.2% calcium hydroxide (Ca(OH)_2_ JCPDS 44-1481), whereas the calcined HAp_900 sample contained 88.3% hydroxyapatite and 11.7% calcium oxide (CaO JCPDS 82-1690). The presence of Ca(OH)_2_ in the as-prepared powder suggests incomplete reaction or residual hydrated calcium species. During wet attritor milling, CaO rapidly hydrates to Ca(OH)_2_, creating a highly alkaline environment. Under these conditions, ammonium ions from diammonium hydrogen phosphate are converted to NH_3_, which volatilizes from the slurry. The alkaline environment favors hydroxyapatite precipitation; however, incomplete mechanochemical reaction can leave residual Ca(OH)_2_, as observed in the as-milled sample. Similar observations have been reported in previous studies, where Ca(OH)_2_ formation was attributed to the hydration of CaO caused by moisture absorption from the surrounding atmosphere [47].

Heat treatment promotes the completion of the solid-state reaction and eliminates the residual hydroxide phase.

The presence of CaO after sintering at 900 °C is consistent with the reported thermal decomposition behavior of Ca(OH)_2_ in the literature and with the XRD observations by the following reaction:

$$\mathrm{Ca}(\mathrm{OH}{)}_{2}\;(s)\to \mathrm{CaO}(s)+H_{2}O\;(g)$$

However, as TGA analysis was not performed in the present study, this interpretation should be regarded as a plausible explanation rather than direct experimental confirmation.

The decrease in the relative HAp content after calcination is more likely related to secondary phase formation and thermal phase evolution than to simple purification. While previous studies have reported that hydroxyapatite may decompose into α-TCP or β-TCP at elevated temperatures [48], no secondary calcium phosphate phases were identified in the present study after calcination at 900 °C. The thermal stability of hydroxyapatite reported in the literature is not entirely uniform. While some authors observed partial decomposition of HAp at elevated temperatures, others reported no significant changes in the XRD patterns over a wide temperature range [49].

In the present case, the XRD patterns showed sharper HAp reflections, indicating improved crystallinity, together with the formation of CaO, which suggests that the heat treatment mainly affected residual non-apatitic calcium phases rather than the hydroxyapatite structure itself. In addition, phase identification using Diffrac^plus^ EVA software showed that the reflections of the heat-treated sample matched well with the standard pattern of hexagonal hydroxyapatite, indicating improved structural ordering after calcination.

### 3.2. Fourier Transform Infrared Spectroscopy (FTIR)

**Figure 3:**
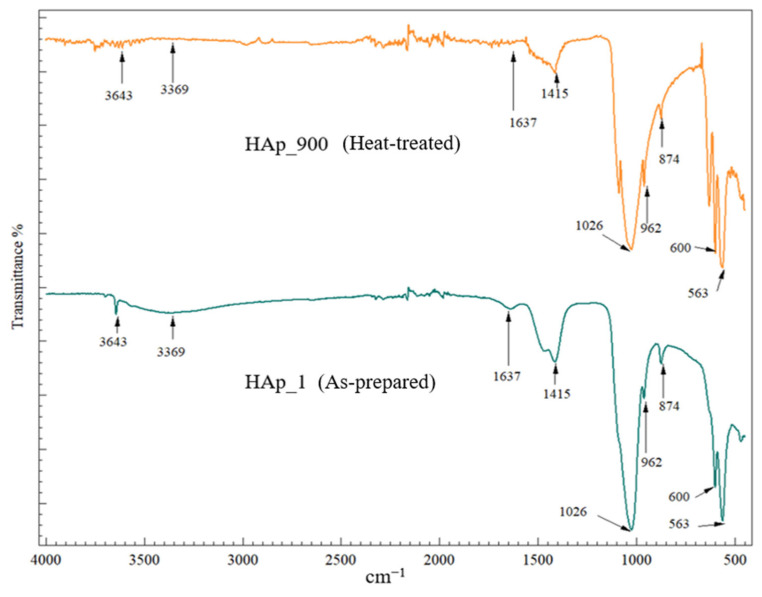
FT-IR spectra of the as-prepared HAp and the HAp after calcination.

As shown in Figure 3, the FTIR spectra of HAp_1 and HAp_900 exhibit the characteristic vibrational features of a phosphate-containing apatite phase, although clear differences can be observed in band intensity and resolution after calcination. The functional groups detected on HAp_1 and HAp_900 are shown in Table 3. In both samples, the bands in the low-wavenumber region around 563 and 600 cm^−1^ were assigned to the *ν*_4_ vibrational mode of PO_4_^3−^. The major peak at 1026 cm^−1^ is consistent with the *v*_3_ antisymmetric stretch vibrations of PO_4_^3−^ bonds [50]. The *v*_1_ symmetric stretch vibrations of PO_4_^3−^ were formed at 962 cm^−1^ [51]. Both of them confirm the formation of an apatite-type calcium phosphate structure [52]. The calcined sample shows sharper and better-resolved phosphate bands than the as-prepared sample, suggesting improved structural ordering and increased crystallinity after heat treatment, in agreement with the XRD results.

Band observed at 874 cm^−1^ can be attributed to *v*_2_ vibration of CO_3_^2−^ and 1415 cm^−1^ is attributed to asymmetric stretching *v*_3_ vibration band of CO_3_^2–^ species [53], while the band at 1637 cm^−1^ was associated with adsorbed water. It should be noted that the XRD analysis did not provide clear evidence for crystalline carbonate-containing phases. This may be due to the low concentration of carbonate species, their possible amorphous nature, or their incorporation into the apatite structure at a level below the XRD detection limit.

In the as-prepared sample, a low-intensity band around 3369 cm^−1^ was assigned to O-H stretching of adsorbed water. A weak band at 3643 cm^−1^ was observed in the as-prepared sample and became barely detectable after heat treatment. This band can be associated with hydroxyl stretching vibrations [47].

The carbonate-related bands were more pronounced in the as-prepared sample but became significantly weaker after calcination, suggesting the removal of residual carbonate-containing species during heat treatment. This behavior is consistent with the thermal evolution expected for hydroxyapatite prepared from biogenic calcium sources. No additional FTIR bands characteristic of immature calcium phosphate phases or incomplete apatite formation were observed, indicating that hydroxyapatite was the dominant phosphate phase in both samples.

### 3.3. Scanning Electron Microscopy (SEM)

**Figure 4:**
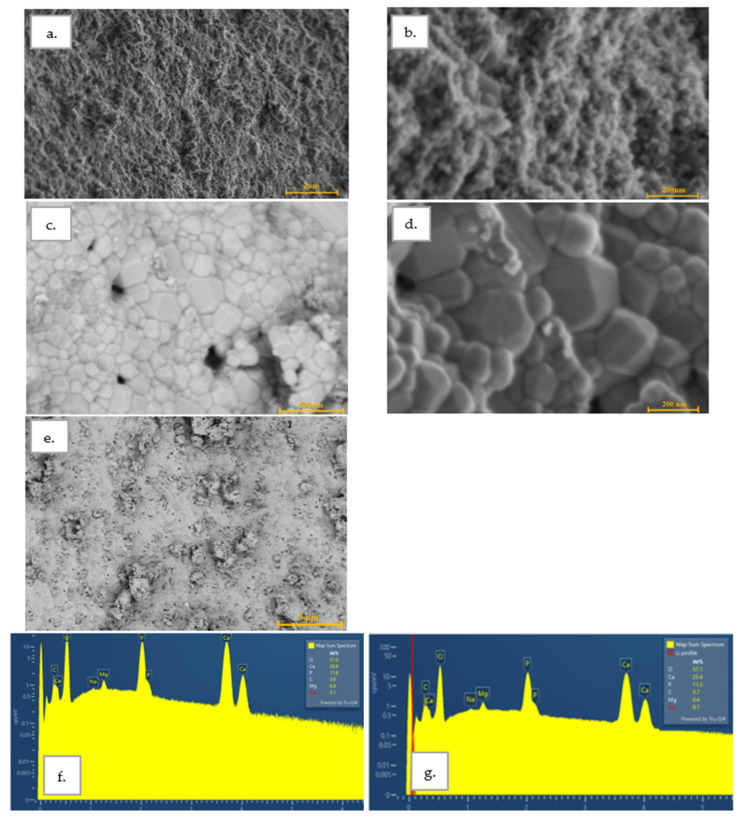
SEM micrographs of the samples: HAp_1 (**a**,**b**), and HAp_900 (**c**–**e**), and EDS spectra of the HAp_1 (**f**) and HAp_900 (**g**).

The SEM analysis revealed a clear morphological evolution of the HAp powders during the process (Figure 4). The as-milled sample (HAp_1; Figure 4a,b) consisted of irregular agglomerates from nanosized particles in the approximate range of 30–100 nm, indicating a fine and highly aggregated structure. After calcination at 900 °C (HAp_900; Figure 4c–e), the powders showed larger and more well-defined particles, with sizes in the range of approximately 100–400 nm, reflecting particle growth and structural coarsening during heat treatment, in agreement with the results of XRD analysis. In some regions, the particles displayed faceted morphologies that are consistent with crystalline hydroxyapatite, which was also confirmed by the XRD results.

Figure 4e also suggests the presence of a macroporous structure with channel-like and seemingly interconnected pores in the size range of approximately 1–5 µm. In addition, the calcined sample showed a rough and textured surface. The interconnected porous structure and rough surface morphology may be advantageous for bone tissue engineering applications, as similar surface characteristics have been reported to support cell attachment and proliferation. Nevertheless, the present study does not include biological evaluation; therefore, these potential benefits remain to be confirmed by future in vitro and in vivo studies [19,54].

EDS analysis of both samples confirmed the presence of Ca, P, O, and C as the major detected elements, while Na and Mg were identified as minor components (Figure 4f). The measured Ca/P molar ratio was 2.28 for the as-milled sample and 2.25 for the calcined sample, both significantly higher than the theoretical stoichiometric value of 1.67 for hydroxyapatite. EDS microanalysis is highly sensitive to experimental parameters, especially electron-beam acceleration voltage and particle-size distribution. The literature shows that variations in these parameters, along with matrix effects, can significantly alter measured Ca and P concentrations [55]. The Ca/P ratios obtained by SEM-EDS should be regarded as semi-quantitative estimates because the technique is sensitive to surface morphology, local compositional heterogeneity, and interaction volume. Consequently, the reported values are used for relative comparison between samples analyzed under identical experimental conditions rather than for determining the absolute bulk Ca/P stoichiometry.

Furthermore, elemental mapping indicated a relatively homogeneous distribution of the detected elements across the analyzed area.

## 4. Conclusions

This study demonstrated a simple and cost-effective route for the synthesis of hydroxyapatite via attritor milling, using diammonium hydrogen phosphate as the phosphate precursor and chicken eggshells as the calcium source. The results show that this approach is effective in producing nanosized hydroxyapatite with high phase purity. SEM and XRD analyses confirmed that the as-milled powders consisted of nanoscale particles with a hydroxyapatite phase purity of 94.8%. After calcination, the samples exhibited a more defined faceted morphology and a porous structure, while the phase purity slightly decreased to 88.3%, as determined by XRD analysis and the Debye-Scherrer method. SEM-EDS analysis indicated the presence of biologically relevant ionic substitutions, including Na and Mg, in both samples, which may contribute to their potential bioactivity. Overall, the calcined hydroxyapatite exhibited a more stable and well-developed structure. The calcined hydroxyapatite exhibited higher crystallinity, improved particle morphology, and enhanced structural stability compared with the as-milled powder. These characteristics are consistent with those reported for hydroxyapatite intended for biomedical applications. Nevertheless, the present study was limited to physicochemical characterization, and further in vitro and in vivo investigations are required to assess its biological performance and suitability for bone tissue engineering.

## Figures and Tables

**Table 1:** Parameters of the synthesized samples.

Sample	Raw Material	Synthesis Method	Rotational Speed (RPM)	Milling Time (h)	Sintering Temperature (°C)	Holding Time (h)
HAp_1	Eggshell	Attritor	2000	5	900	-
HAp_900	Eggshell	Attritor	2000	5	900	1

**Table 2:** Components of the samples and crystallite size (D) of the hydroxyapatite.

Sample	HAp %	HAp D (Å)	Ca(OH)_2_ %	CaO %
HAp_1	94.8	123	5.2	0
HAp_900	88.3	560	0	11.7

**Table 3:** Comparison of FTIR spectra of the samples.

Wavenumber (cm^−1^)	Functional Group	Appearance in HAp_1	Appearance in HAp_900
3643	O-H Stretching	Distinct, sharp peak	Significantly reduced
3369	Adsorbed H_2_O	Broad, shallow band	Decreased intensity
1637	Adsorbed H_2_O	Weak band	Reduced intensity
1415	CO_3_^2−^	Medium, broad band	Present, reduced intensity
1026	PO_4_^3−^ Asymmetric stretching	Strong, broad peak	Strong, sharp peak
962	PO_4_^3−^ Symmetric stretching	Medium-intensity peak	Sharp, distinct peak
874	CO_3_^2−^	Medium, clear peak	Present, lower intensity
600	PO_4_^3−^ Bending vibration	Sharp peak	Very sharp, part of a phosphate doublet
563	PO_4_^3−^ Bending vibration	Sharp peak	Very sharp, well-resolved

## Data Availability

The original contributions presented in this study are included in the article. Further inquiries can be directed to the corresponding author.
